# EHMTI-0248. Evaluation of headache prevalence and characteristics in orphans aged 12-17

**DOI:** 10.1186/1129-2377-15-S1-B34

**Published:** 2014-09-18

**Authors:** A Ates Senturk, B Alpaslan, U Uygunoglu, D Uluduz, S Saip, A Siva

**Affiliations:** 1 Neurology Department, Istanbul University Cerrahpasa Medical Faculty, Istanbul, Turkey

## Introduction

The epidemiological studies show that presence of divorced parents or loss of a parent is a risk factor for headache during childhood.

## Aims

We aimed to investigate the frequency, clinical features, trigger factors, comorbidities, effects on daily life and accompanying emotional and behavioral problems of the migraine and tension-type headache(TTH) in the orphan children.

## Methods

We performed our study in a school where the orphan children study.415 students of 12-17 age group are counted in the study.PedMIDAS scale of 5 questions and strength and difficulties questionnaire of 25 questions are asked to fill. The results are compared by dividing the children into 2 groups of 12-14 and 15-17 age groups.

## Results

In the 12-14 age group, the frequency of primary headache is found as 59,8% with the frequency of migraine is 24,4% and the frequency of TTH is 35,4%. In the 15-17 age group the frequency of headache is 59,7% and 23,6% of them were composed of migraine where as the 36,1% of them fulfil the criteria of TTH.

## Conclusions

Stressful life events during childhood such as parent loss is found to be relevant with becoming chronic and higher frequency of headache.

No conflict of interest.

